# DNA methylation landscape in pregnancy-induced hypertension: progress and challenges

**DOI:** 10.1186/s12958-024-01248-0

**Published:** 2024-07-08

**Authors:** Fengying Deng, Jiahui Lei, Junlan Qiu, Chenxuan Zhao, Xietong Wang, Min Li, Miao Sun, Meihua Zhang, Qinqin Gao

**Affiliations:** 1 Key Laboratory of Maternal & Fetal Medicine of National Health Commission of China, Shandong Provincial Maternal and Child Health Care Hospital Affiliated to Qingdao University, Jinan, 250014 China; 2 Institute for Fetology, the First Affiliated Hospital of Soochow University, Suzhou, 215006 P. R. China; 3 Department of Oncology and Hematology, Suzhou Hospital, Affiliated Hospital of Medical School, Nanjing University, Suzhou, Jiangsu 215153 P.R. China; 4 Department of Obstetrics and Gynecology, the First Affiliated Hospital of Soochow University, Suzhou, 215006 P. R. China

**Keywords:** Pregnancy-induced hypertension, DNA methylation, Prediction, Diagnosis and Treatment

## Abstract

Gestational hypertension (PIH), especially pre-eclampsia (PE), is a common complication of pregnancy. This condition poses significant risks to the health of both the mother and the fetus. Emerging evidence suggests that epigenetic modifications, particularly DNA methylation, may play a role in initiating the earliest pathophysiology of PIH. This article describes the relationship between DNA methylation and placental trophoblast function, genes associated with the placental microenvironment, the placental vascular system, and maternal blood and vascular function, abnormalities of umbilical cord blood and vascular function in the onset and progression of PIH, as well as changes in DNA methylation in the progeny of PIH, in terms of maternal, fetal, and offspring. We also explore the latest research on DNA methylation-based early detection, diagnosis and potential therapeutic strategies for PIH. This will enable the field of DNA methylation research to continue to enhance our understanding of the epigenetic regulation of PIH genes and identify potential therapeutic targets.

## Introduction

Pregnancy-induced hypertension (PIH), the most common medical syndrome in pregnancy, is further categorized into four subtypes based on potential differences in pathogenesis and pregnancy outcomes: gestational hypertension, chronic hypertension, preeclampsia (PE), and PE with coexisting chronic hypertension [1–3]. PIH not only adversely affects pregnant women and fetuses/newborns, but also increases the risk of various chronic diseases, including cardiovascular disease and metabolic dysfunction, in both mother and offspring [4–7]. Therefore, elucidating the pathological mechanisms and developing effective therapeutics for PIH remains a priority. Accumulated findings have demonstrated that epigenetic modifications, particularly DNA methylation, relate to heritable changes in placental gene expression that contribute to the development of placental dysfunction and are a critical mediator in the onset of PIH [8, 9]. It is therefore believed that understanding the DNA methylation mechanisms underlying the initiation of PIH may contribute to the identification of novel therapeutic targets for PIH treatment, thereby improving fetal growth and reducing the risk of chronic disease in future generations.

**Fig. 1:**
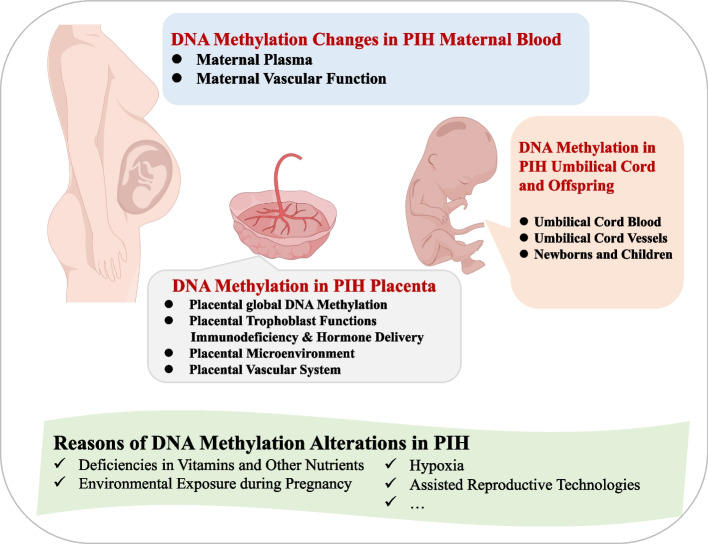
The outline of this review

DNA methylation, a stable epigenetic modification of DNA, involves the addition of a methyl group to cytosine in CpG-rich regions of the genome. This process is facilitated by DNA methyltransferase (DNMT), with S-adenosyl-methionine (SAM) acting as the methyl donor. DNMTs can be divided into two primary categories: de novo DNMTs (DNMT3a/3b/3L) and maintenance DNMTs (DNMT1/2) [10]. During active DNA demethylation, an intermediate modification called 5-hydroxymethylcytosine (5hmC) is formed, catalyzed by the ten-eleven translocation (TET1-3) proteins [11]. These enzymes play a pivotal role in embryonic development, establishing, maintaining, and dynamically regulating the somatic gene methylation pattern during embryogenesis. Abnormal DNA methylation has been observed in cells from various sources in individuals with PIH. Researchers have analyzed not only placental cells but also maternal blood cells, cell-free DNA, and umbilical cord blood leukocytes from fetuses. In this review, we present a succinct overview of the existing literature and perform a comprehensive assessment of the involvement of abnormal DNA methylation in the pathogenesis of PIH (Fig. 1). Additionally, we explore the potential utility of DNA methylation markers as predictive biomarkers for the onset and severity of PIH. Furthermore, we outline current investigations focusing on the application of DNA methylation-based approaches for the prevention, diagnosis, and potential therapeutic interventions for PIH.

## DNA methylation in PIH placenta

Although the exact pathogenesis of PIH remains unclear, the placenta is believed to play a key role, and DNA methylation may be associated with altered placental function in PIH progression. This section presents abnormal DNA methylation in PIH placenta, as well as a focus on describing the effects of DNA methylation on placental trophoblast and vascular function.

### Global DNA methylation levels in PIH placenta

In recent years, researchers have investigated global DNA methylation in the PIH placentas. Jia et al. analyzed human PE placentas and found 296 genes with significantly aberrant DNA methylation [12]. Blair et al. found significant DNA methylation alterations at 38,840 CpG sites in whole chorionic villus samples from early onset pre-eclampsia (EOPE) placentas, most of which (74.5%) were hypomethylated in EOPE [13]. Xu et al. identified 536 upregulated genes with hypomethylation and 322 downregulated genes with hypermethylation in the PE placentas. Gene ontology analysis showed that these genes were primarily involved in processes such as mitotic cell cycle, calcium homeostasis, and cell proliferation [14]. Although the candidate genes in these studies are distinct, differences in their DNA methylation provide evidence that placental DNA methylation is altered during the pathophysiological course of PIH, and the identification of placental gene-specific methylation profiles may provide valuable insights into pathways that may be epigenetically regulated.

As the severity of PE symptoms can vary among pregnant women due to the disease's heterogeneity, Lim et al. investigated the genome-wide DNA methylation profile of PE placentas based on severity characteristics. They found that DNA hypomethylation was widespread in mild PE compared to PE with severe features. In PE with severe features, hypermethylated CpG sites showed physiopathological relevance [15]. Furthermore, the total amount of DNA methylation in the placenta has been observed to strongly correlate with the gestational age. Li et al. discovered that placental DNA methylation levels were higher in PE and premature birth compared to term birth. The effects of gestational age were ruled out, and a total of 808 differential methylation positions were discovered, the majority of which were hypomethylated in PE placentas [16]. Benjamin et al. reported that dynamic DNA methylation changes in the human placenta can be used to predict gestational age. They developed an accurate tool for predicting the gestational age of placentas and found that EOPE is associated with placental aging [17]. In addition, Chu et al. found evidence that fetal gender influences DNA methylation at autosomal loci in PE placentas [18]. Placenta development and invasion, both of which are influenced by the cytoskeleton and migration, occur more slowly in female fetuses compared to male fetuses [19, 20]. Male placentas have a greater capability for nutrient transfer than female placentas, and their functions throughout pregnancy are distinct [19].

**Fig. 2:**
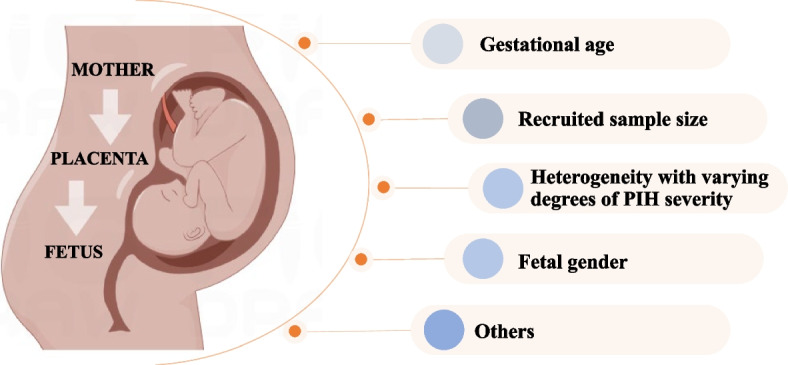
Possible factors affecting the results of global DNA methylation levels in PIH placenta

Taken together, it is undeniable that patients with PIH experience dysregulation of placental DNA methylation. It is possible that maternal and fetal genes have independent or interactive impacts on the risk of PIH, and the disease exhibits heterogeneity with varying degrees of severity. Therefore, future studies analyzing placental global DNA methylation should carefully consider PIH's heterogeneity, gestational age, and fetal gender (Fig. 2).

### DNA methylation and placental trophoblast functions

Different types of trophoblast cells have been identified in the placenta, including cytotrophoblast (CTB), syncytiotrophoblast (STB) and extravilloustrophoblast (EVT). These different cell types work together to perform various tasks, such as forming chemical and physical barriers, producing hormones, and maintaining immune tolerance. It is evident that trophoblast malfunctions are linked with various pregnancy disorders and pose a significant threat to the well-being of both the mother and the fetus. Numerous studies have examined placental trophoblast malfunction in PIH from a DNA methylation perspective, focusing on immune deficiency and hormone delivery.

#### DNA methylation and placental immunodysfunction

During the first trimester of pregnancy, there is a significant increase in innate immune activity, which facilitates the implantation and growth of the embryo and the placenta [21]. Immune dysregulation during implantation can lead to a lack of trophoblast invasion, resulting in a shallow arterial remodeling of the spiraled arteries in the myometrium. This, in turn, leads to inadequate placental perfusion and eventually placental ischemia [3]. Recent studies have reported that DNA methylation changes within many immune-related genes impair the placental trophectoderm, which impedes the development of maternal–fetal immune tolerance and contributes to PIH (Table 1).

**Table 1:** DNA methylation changes in PIH involved in the regulation of placental immunodeficiency and placental vascular system

Sample Size	Sample Type	DNA methylation	Target gene	Gene expression	Functions	Ref
3	villi	Hypermethylation	Syncytin-1	Decreased	Inhibit trophoblast cell fusion	[25]
16	placental bed	Hypomethylation	TF	Increased	Hinder the development of placental immune tolerance	[28]
19	villi	Hypomethylation	Galectin 13/14/16	Increased	Decrease immune tolerance at the mother-fetus interface	[32]
19	placental bed	Hypermethylation	HLA-G	Decreased	Reduce maternal immune tolerance in fetuses	[39]
5	placental bed	Hypermethylation	HLA-DPB1, HLA-DRB1	Decreased	Cause abnormalities in interferon γ-mediated signaling pathways	[15]
47	placental bed	Hypermethylation	ALCAM	Decreased	Reduce trophoblast viability and invasiveness	[115]
20	placental bed	Hypomethylation	ENTPD1, ZDHHC14	Decreased	Inhibition of trophoblast proliferation and invasion	[41]
93	placental bed	Hypomethylation	VEGF	Increased	Disruption of angiogenesis leads to placental insufficiency and endothelial dysfunction	[131]
11	placental bed	Hypomethylation	MMP9	Increased	Blocking adhesion molecule-mediated invasion of EVT	[138]
48	placental bed	Hypomethylation	TIMP3	Increased	Reduce trophoblast invasiveness	[141]
37	placental bed	Hypomethylation	ADAMTS7	Increased	Inhibition of migration and invasion of trophoblasts	[144]
40	placental bed	Hypermethylation	AVPR1A	Decreased	Weakening of placental vasoconstriction	[147]
42	placental bed	Hypermethylation	OXTR	Decreased	Weakening of placental vasoconstriction	[150]
8	placental bed	Hypermethylation	NOX5	Decreased	Attenuates cell proliferation and differentiation	[91]

*TF* Tissue Factor, *HLA* Human Leukocyte Antigens, *ALCAM* Activated Leukocyte Cell Adhesion Molecul, *ENTPD1* CD39/ectonucleoside triphosphate diphosphohydrolase-1, *ZDHHC14* Zinc finger DHHC-type containing 14, *VEGF* Vascular Endothelial Growth Factor, *PIGF* Placental Growth Factor, *sFlt-1* soluble FMS-like tyrosine kinase-1, *MMP* Matrix Metalloproteinase, *EVT* Extravillous trophoblast, *TIMP* Tissue Inhibitors of Metalloproteinase, *ADAMTS* a disintegrin and metalloproteinase with thrombospondin motif, *AVPR* Arginine Vasopressin Receptor, *OXTR* Oxytocin Receptor, *NOX* NADPH Oxidases

Syncytins are a group of membrane glycoproteins. Syncytin-1 is mainly expressed by trophoblast cells, and mediates the fusion of trophoblasts to form STB. It has been reported to participate in regulating inflammation, anti-apoptosis, and immune suppression in PE placentas [22, 23]. Decreased expression of syncytin-1 has been observed in PE placentas, and this decrease has been linked to hypermethylation of its gene promoter [24–26]. Ruebner et al. found that overexpression of DNMT3a could be responsible for decreased syncytin-1 expression through promoter hypermethylation, which in turn inhibits trophoblast cell fusion [25]. Based on these findings, it is highly speculated that a broad aberrant DNA methylation pattern within the syncytin-1 gene downregulates its expression, which participates in the pathogenesis of PIH potentially by affecting trophoblast immune and endocrine functions.

Tissue factor (TF) is a membrane glycoprotein that plays a crucial role in fetal and placental damage in mouse models of PE and is involved in the etiology of PE [27, 28]. Liu et al. discovered that TF expression was increased in human PE placentas, and the TF gene promoter had significantly less DNA methylation [28]. The stable and high expression of the TF gene is essential for regulating the development and function of regulatory T (Treg) cells [29, 30], and its presence and relative stability are crucial for ensuring appropriate maternal–fetal tolerance. Consequently, aberrant TF expression in the placenta may lead to the loss of immunosuppressive function, hindering the development of placental immunological tolerance.

Galectins are highly expressed at the maternal–fetal interface and act as key regulator proteins of the immune response in maternal–fetal immune tolerance [31]. Three genes in a Chr19 cluster, encoding galectin 13/14/16, primarily confer immune tolerance in maternal–fetal interactions. Than et al. reported that the low DNA methylation of differentially methylated regions (DMRs) surrounding their transcription start sites may enable the activated transcription of these genes [32]. The dysregulation of these galectin genes in the PE placenta due to DNA methylation can affect immune tolerance at the maternal–fetal interface.

Human leukocyte antigens (HLA) molecules are highly correlated with mother's ability to carry and deliver a healthy allogeneic fetus. Immune tolerance between mother and fetus is partially mediated by HLA, with trophoblast cells expressing HLA-C, non-classical HLA-Ib molecules (HLA-E, -F, and -G), and HLA class II (HLA-DRB1, and -DPB1) antigens [33]. In physiological conditions, HLA-G, which is abundantly and specifically expressed on EVTs, promotes fetal-maternal immune tolerance by reducing NK cell toxicity and cytokine production [34, 35]. Soluble HLA-G also has a regional effect on spiral artery remodeling in the uterus [36]. The significance of HLA-G is further emphasized by the occurrence of PE when its expression at the maternal–fetal interface is abnormal [37, 38]. Several studies have reported that promoter DNA methylation influences HLA-G expression [39, 40]. Tang et al. observed extremely high levels of CpG methylation in the promoter region of dysregulated HLA-G in PE placentas, and demonstrated that DNMT1-mediated promoter hypermethylation of HLA-G is associated with PE [39]. In addition to HLA-G, HLA-DPB1 and HLA-DRB1 have been found to be involved in the interferon γ-mediated signaling pathway cluster specific to PE. Severe PE (SPE) cases show increased promoter DNA methylation of HLA-DPB1 and HLA-DRB1 compared to general PE cases. This difference in DNA methylation levels may indicate a relationship between vulnerability to illness and interferon γ-mediated signaling pathways in SPE [15].

In addition to the major molecules mentioned above, several genes are involved in immune tolerance at the maternal–fetal interface and exhibit altered expression in PE placentas due to DNA methylation modifications. For instance, CD39/ectonucleoside triphosphate diphosphohydrolase-1 (ENTPD1) and zinc finger DHHC-type containing 14 (ZDHHC14) have been found to have promoter hypomethylation in PE placentas, resulting in reduced expression of these genes [41]. ENTPD1, which is the primary ectonucleotidase in fetal trophoblast tissue, is also a surface marker of Treg cells. Reduced expression of ENTPD1 could contributes to the development of clinical characteristics associated with PE [42].

It is evident that abnormalities in trophoblast cell proliferation, migration, invasion, and adhesion during the first trimester of pregnancy result from placental immunodeficiency, which significantly raises the risk of PE. The DNA methylation perspective enhances our understanding of the pathogenesis; however, there is still a substantial knowledge gap, and further studies are necessary to establish the link between DNA methylation and the progression of PIH.

#### DNA methylation and placental hormone regulation

Glucocorticoids, which belong to the class of steroid hormones, participate in reproduction, including the initiation of pregnancy, successful fetal development, and delivery. Therefore, maintaining appropriate levels of circulating glucocorticoids in the fetus is vital for ensuring normal growth and development within the uterus. The primary bioavailable glucocorticoid is cortisol. It is believed that the enzyme 11β-hydroxysteroid dehydrogenase 2 (11βHSD2) is responsible for protecting the fetus from excessive exposure to maternal glucocorticoids by converting active cortisol into an inactive form [43]. This section aims to summarize whether abnormalities in the glucocorticoid receptor (nuclear receptor subfamily 3, group C, member 1, NR3C1) and 11βHSD2 are involved in the mechanism of PIH occurrence and whether this process is mediated by DNA methylation.

NR3C1 is responsible for the effects of cortisol, and the cortisol-NR3C1 complex can bind to glucocorticoid response elements in the promoters of target genes [44]. NR3C1 expression partially determines the impact of cortisol on blood pressure [45]. A lack of NR3C1 expression in the placenta has negative consequences for fetal health and growth [46]. When BeWo and JEG3 choriocarcinoma cells were exposed to the DNMT1 inhibitor 5-Aza-CdR, significant reductions in promoter DNA methylation of the NR3C1 gene were observed [47]. Evidence suggests that EOPE is associated with an increase in DNA methylation levels at CpG sites of the NR3C1 gene [48]. Notably, Putra et al. observed different patterns of NR3C1 promoter methylation in normotensive, hypertensive, and hypotensive pregnancies and found evidence of an independent association between placental NR3C1 proximal promoter methylation and maternal blood pressure [49]. This independent link between placental NR3C1 gene promoter methylation and maternal blood pressure was supported by data from other studies [48, 50]. It is without a doubt that differential methylation within the NR3C1 gene in placentas is a likely pathological mechanism in PIH.

Placenta contains a barrier against glucocorticoids, which is formed by 11βHSD2. Due to its high affinity for cortisol, 11βHSD2 acts as a placental glucocorticoid barrier, safeguarding the developing fetus from excess glucocorticoids. Disruption of the barrier has been linked to pregnancy complications and the future onset of chronic diseases [51–53]. Several studies have reported that the glucocorticoid barrier is damaged in PE placentas [54, 55], accompanied by the increased cortisol levels [56]. SCHOOF et al. found that in the European population (*n* = 18), 11βHSD2 is downregulated in PE placenta [57]. It is known that 11βHSD2 expression is tightly regulated by DNA methylation [58, 59]. However, Hu et al. found that in the Chinese population (*n* = 10), the DNA methylation level of the 11βHSD2 gene promoter region in the PE placenta did not change [60]. Current studies on epigenetic alterations of the 11βHSD2 gene in PE are limited and may yield unreliable results due to variations in sample size or population ethnicity.

The placenta plays a significant role in releasing hormones such as progesterone, and placental lactogen, which are crucial for normal pregnancy progression. However, there is currently a lack of studies comparing DNA methylation alterations in placental secretion function between PIH and normal pregnancies. This article aims to serve as a catalyst for further research into the role of DNA methylation in PIH-related placental insufficiency and hopes to inspire academics to investigate this topic.

### DNA methylation and placental microenvironment

#### DNA methylation and imprinted genes

**Fig. 3:**
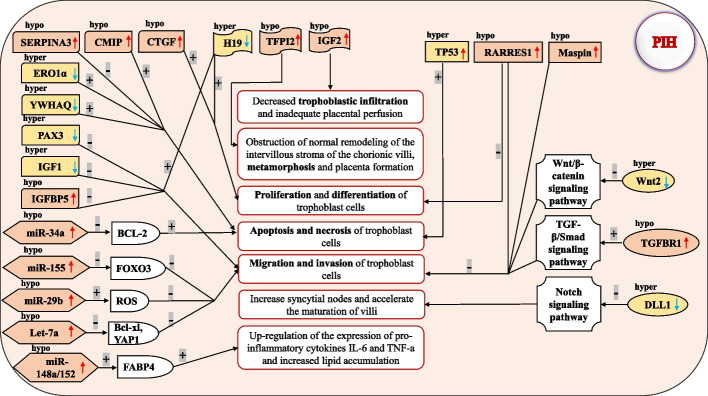
The occurrence of PIH is associated with differential DNA methylation of placental genes. This figure illustrates the genes in the placenta that contribute to PIH due to variations in DNA methylation. These genes include imprinted genes, tumor suppressor genes, placental classical signaling pathways, placental microRNAs, and several other protein genes. Abbreviation: hyper, hypermethylation; hypo, hypomethylation; + , promote; -, inhibit; TFPI2, tissue factor pathway inhibitor 2; VHL, von Hippel Lindau tumor suppressor; RARRES1, retinoic acid receptor responder 1; TGF, transforming growth factor; DLL1, Delta-like 1; BCL-2, B-cell CLL/lymphoma 2; FABP4, fatty acid binding protein 4; ROS, reactive oxygen species; Bcl-xl, B-cell lymphoma-extra large; YAP1, Yes-associated protein 1; FOXO3, Forkhead-box O3; IGFBP5, insulin-like growth factor-binding protein 5; IGF, insulin-like growth factor; PAX3, Paired Box 3; YWHAQ, Tyrosine 3-monooxygenase/tryptophan 5-monooxygenase activation protein, theta polypeptide; ERO1α, endoplasmic reticulum oxidase 1α; SERPINA3, serpin peptidase inhibitor clade A member 3; CMIP, C-Maf inducing protein; CTGF, connective tissue growth factor

Epigenetic reprogramming, including genomic imprinting, occurs during germ cell development and continues into pre-implantation stages. The expression of imprinted genes is regulated by DNA methylation, which differs between parents. Imprinted genes, genes that are exclusively expressed from one allele-linked to the parental origin of these allele-, are expressed in the placenta. Some are expressed from the paternal allele, while other are expressed from the maternal allele, which serves as a connection between the mother and the developing baby. The human placenta contains a significant number of placenta-specific imprinted transcripts [61, 62]. Many genes in the placenta exhibit parent-of-origin specific imprinting, which may contribute to processes such as trophoblast proliferation, differentiation, angiogenesis, and nutrient transport [63]. Recent studies have reported associations between certain imprinted genes and PIH occurrence [64–66] (Fig. 3). These findings enhance our understanding of the pathogenesis of PIH.

Insulin-like growth factor 2 (IGF2), which is maternally imprinted and paternally expressed, plays a role in promoting placental growth and transport capacity by regulating the fetus's placental supply and the genetic demand for maternal nutrition [67, 68]. Berg et al. found that patients with EOPE exhibited altered DNA methylation of the IGF2 DMRs in their placental tissue, umbilical cord leukocytes, and vein endothelial cells, but not in those with late-onset preeclampsia (LOPE) [69]. He et al. discovered that the DNA methylation levels of IGF2 DMRs were significantly lower in the umbilical cord blood of infants born to PE. This suggests that reduced IGF2 DMRs methylation is associated with maternal PE and health problems in later life [70]. Furthermore, the average DNA methylation level at IGF2 DMRs was significantly correlated with PE, even after adjusting for birth weight, maternal age, gestational age at delivery, and fetal gender [70]. Insulin-like growth factor-binding protein 5 (IGFBP5) belongs to the IGFBP family of proteins, inhibits the migration of trophoblast cells induced by insulin-like growth factor-1 (IGF1) and IGF2 [71]. Jia et al. identified IGFBP5 as a critical downstream factor of DNMT3A. They discovered that downregulating DNMT3A in the several trophoblasts cell lines increases IGFBP5 expression by decreasing its promoter methylation, consequently suppressing trophoblast cell migration and invasion [72]. IGFBP5 is a secreted protein, and as such, it makes it a promising biomarker. Ma et al. reported that the IGF1 gene promoter is highly methylated in PE placentas, and these placentas have much lower levels of IGF1, moreover, PE is associated with hypermethylation of IGF1 promoter mediated by DNMT1[73].

The H19 exhibits high expression in invasive intermediate trophoblast cells and villous CTB, but its expression is barely detectable in STB [74, 75]. The variable expression pattern of H19 is consistent with its role in controlling trophoblast invasiveness, providing further evidence that the H19 gene is essential for early placental development. Gao et al. found that the overall DNA methylation levels within the H19 gene were considerably higher in EOPE placentas. Meanwhile, hypermethylation in the H19 gene promoter region was correlated with lower expression of H19 gene [65]. H19 exon 1 represents the longest functional nuclear portion of the H19 gene. Placental tissues from women with SPE exhibited lower DNA methylation levels in H19 exon 1[76]. After treatment with the methylation inhibitor 5-Aza-Dc, trophoblast methylation levels significantly decreased, H19 gene mRNA levels increased, and trophoblast cell proliferation, migration, and invasion were dramatically slowed down [76]. Additionally, the incidence of PE was lower in individuals with suppressed H19 mRNA expression due to promoter hypermethylation [77]. Recent clinical trials have examined the effectiveness of metformin in the treatment and prevention of PE [78]. These trials have shown that metformin can affect H19 gene DNA methylation, resulting in changes in H19 expression. The therapeutic benefit of metformin in PE is associated with a dose-dependent increase in H19 methylation and suppression of H19 expression. These studies collectively suggest that hypermethylation at specific CpG sites within the H19 gene may contribute to trophoblast dysfunction and the pathogenesis of PE. However, further extensive and carefully designed investigations in diverse racial and ethnic groups are necessary to validate these findings.

As a member of an imprinted gene cluster, tissue factor pathway inhibitor 2 (TFPI2) acts as an inhibitor of Kunitz-type serine proteinases. In PE placentas, TFPI2 expression is upregulated, which hinders proper villous interstitial remodeling, decidualization, and placental formation [79, 80]. Xiao et al. suggested that the epigenetic mechanism of TFPI2 may be involved in the pathogenesis of PE, and they found significantly lower levels of DNA methylation of the TFPI2 gene promoter in PE placentas compared to controls [80].

In summary, aberrant DNA methylation in imprinted genes such as H19, IGF2, and TFPI2 is associated with an increased risk of PIH. Disruptions in imprinted gene expression can result in phenotypic disorders in humans, as they play a crucial role in regulating growth and development during the embryonic and postnatal stages. Nonetheless, several unanswered questions remain, including the interaction between additional paternal or maternal imprinted genes and DNA methylation as well as their impact on trophoblast cells and placental function.

#### DNA methylation and tumor suppressor genes

The formation of the placenta, which is unique to mammals, can recapitulate many cancer features. The processes of placentation and tumorigenesis involve similar physiological events, such as rapid cell proliferation and invasion of surrounding tissues. The invasive behavior of cancer cells and placental trophoblast cells, as well as the role of aberrant DNA methylation, are well-known resemblances. The contribution of tumor suppressor genes in cancer is well-documented. Many tumor suppressor genes are expressed in the placenta, and also play a crucial role in placentation and the maintenance of trophoblast functions (Fig. 3).

During pregnancy, the tumor suppressor gene maspin plays a crucial role in controlling trophoblast invasion by being variably expressed in the human placenta. The presence of recombinant maspin greatly reduces the invasion of trophoblasts [81]. Increased maspin expression was observed in the PE placentas when DNA methylation levels in the maspin gene promoter region were low, suggesting that DNA methylation plays a role in the regulation of maspin expression and affects trophoblast invasion, contributing to the pathogenesis of PE [82]. Normal placental development depends on the von Hippel Lindau tumor suppressor (VHL) protein, which is under-expressed in EOPE placentas [83]. Alahari et al. shown that reduced methylation in the CpG-rich area of the VHL gene promoter facilitates VHL binding to E2F4, a transcriptional inhibitor, thereby inhibiting VHL transcription. Subsequently, the absence of VHL leads to an increase in hypoxia inducible factor (HIF) 1A, which disrupts the oxygen homeostasis characteristic of EOPE placentas [83]. This study provides the first evidence that promoter hypomethylation of the VHL gene at a crucial transcription factor binding site may be involved in the pathogenesis of EOPE. Previously, retinoic acid receptor responder 1 (RARRES1) was considered a tumor suppressor gene that is downregulated in cancer cells and promotes apoptosis [84, 85]. However, RARRES1 is under-expressed in most placental cells, while its expression is upregulated in CTB and STB cells due to promoter demethylation. The increase in RARRES1 in PE placentas may be attributed to disturbed trophoblast development, and RARRES1 may impede the invasion and migration of terminal placental EVTs [86, 87].

TP53 has been found to have implications for the life cycle of placental trophoblast cells. There is a 5.6-fold increase in the mRNA expression of the TP53 gene in PE placentas compared to control placentas. For the correlation, there is a strong trend toward a higher expression in methylated conditions (*p* = 0.06) [88]. Another tumor suppressor gene, bladder cancer-associated protein (BLCAP), has been found to be significantly expressed in the placenta of women with perinatal ectopic pregnancies. Interestingly, the promoter methylation level of BLCAP is significantly lowered in the PE placentas [16]. However, it is still unclear whether DNA methylation-mediated BLCAP expression has any effect on the progression of PE, and further investigation is needed in this area.

In the placenta, DNA methylation changes in tumor suppressor genes may impact trophoblast cell proliferation, apoptosis, migration, invasion, and adhesion, ultimately leading to a wide range of clinical symptoms associated with PIH. There are still many tumor suppressor genes that have not been thoroughly explored, especially in relation to DNA methylation in the context of PIH pregnancy.

#### DNA methylation and placental classical signaling pathways

The Wnt2 protein is a glycoprotein that interacts with the human brown fly receptor to activate the Wnt/β-catenin signaling pathway, resulting in the expression of target genes. In Wnt2 knockout mice, reduced placental vascularization and structural defects lead to increased fetal mortality due to trophoblastic invasion of the spiral arteries [89]. Methylation of the Wnt2 gene promoter has been observed in SPE placentas, which is associated with decreased Wnt2 gene expression [90, 91]. Liu et al. found a direct correlation between increased methylation of the Wnt2 gene promoter and decreased Wnt2 gene expression. They also demonstrated that DNA methylation plays a role in the regulation of Wnt2 gene expression, leading to trophoblast dysfunction, which in turn affects trophoblast invagination and helical artery remodeling, and thus is involved in the pathophysiology of PE [92]. Wnt3, which plays a crucial role in regulating decidualization, stromal cell proliferation, and trophoblast invasion, has been found to be hypomethylated in its gene promoter in PE placentas. This leads to increased expression that compensates for the low activation status of the Wnt/β-catenin signaling pathway [93].

Additionally, the transforming growth factor β (TGF-β) pathway plays a role in maintaining placental functions and establishing pregnancy. The TGF-β superfamily transmits signals by forming heteromeric complexes of type I and type II transmembrane serine/threonine kinase receptors [94]. Activation of TGF-β receptor 1 (TGFBR1) leads to the phosphorylation of receptor-specific Smad proteins, namely Smad2 and Smad3 [95]. TGF-β pathway exhibits extensive promoter hypomethylation in the PE placentas. This hypomethylation is accompanied by an enrichment of TGF-β pathway-related genes, including SMAD family member 3 (SMAD3), v-ski sarcoma viral oncogene homolog (SKI), runt-related transcription factor 3 (RUNX3), and TGFB-induced factor homeobox 1 (TGIF1) [96]. In the placenta of women with early-onset severe PE (EOSPE), trophoblast cells exhibit elevated levels of TGFBR1 due to downregulation of DNMT3A. Restoring normal trophoblast migration and invasion can be achieved by inhibiting TGFBR1 and TGF-β/Smad signaling. This indicates that DNA hypomethylation-mediated upregulation of the TGFBR1 gene is associated with the development of EOSPE by inhibiting trophoblast migration and invasion [72, 96].

The receptor-ligand Notch signaling family regulates various cellular processes, such as proliferation, apoptosis, invasion, and adhesion. Dysregulation of the Notch signaling pathway has been observed in PE placentas [97, 98]. Shimanuki et al. analyzed the Delta-like 1 (DLL1) gene, which is the Notch activating ligand, in PE placentas. They discovered a higher frequency of DLL1 gene DNA methylation and significantly lower DLL1 expressions in PE placentas[98]. Their analysis also revealed that EOSPE placentas with DLL1-DNA methylation exhibited an increase in syncytial knots and accelerated villous maturation [98]. These findings suggest that altered Notch signaling through DNA methylation may contribute to the pathogenesis of PE.

In conclusion, the Wnt/β-catenin signaling and TGF-β pathways are the most reported signaling pathways involved in the development of PIH. The Notch signaling pathway have also been reported to be associated with PIH (Fig. 3). These findings significantly enhance our understanding of the role of DNA methylation in the pathophysiology of PIH and suggest a potential novel therapeutic approach targeting DNA methylation for this disorder.

#### DNA methylation and placental MicroRNAs

Numerous studies have demonstrated the crucial role of miRNAs in the regulation of placental development and pathology [99, 100]. Previous research has indicated that PE placentas exhibit distinct miRNA expression profiles compared to normal placentas. Differentially expressed miRNAs are characteristic of mild and SPE. In many cases, these miRNAs often target key classical signaling pathways that control trophoblast proliferation and invasion, which are directly implicated in PIH [99, 100]. MiRNAs have also been recognized as potential therapeutic targets and diagnostic tools for PIH.

For instance, miR-34a has been found to regulate somatic cell reprogramming, cell cycle, apoptosis, and metastasis [101]. One study showed that PE placentas exhibits higher expression of pri-miR-34a due to promoter hypomethylation [102]. On the other hand, Guo et al. found significantly higher levels of miR-34a in the PE placentas, and demonstrated that upregulation of miR-34a induces trophoblast apoptosis in PE through inhibition of B-cell CLL/lymphoma 2 (BCL-2) protein expression [103]. Rezaei et al. further elucidated that hypomethylation of the miR-34a promoter increased susceptibility to PE, suggesting that miR-34a may be a target gene for PE development [104].

MiR-29b has been implicated in several studies on PE, where its presence has been found to decrease trophoblast cell proliferation and migration [105, 106]. It has been discovered that placental hypomethylation controls the induction of placental reactive oxygen species (ROS) production by upregulating the expression of miR-29b [107]. The Let-7 family of miRNAs exhibits strong sequence and functional conservation. Zha et al. indicates that let-7a is hypomethylated in EOSPE placentas and let-7a is highly expressed, leading to the downregulation of B-cell lymphoma-extra large (Bcl-xl) and Yes-associated protein 1 (YAP1), which decreases trophoblast invasion while promoting cell death [108]. Thus, elevated let-7a expression causes JEG-3 cells to become less viable, halts the cell cycle, and induces apoptosis [108, 109]. Luo et al. found that miR-155 showed significant up-regulation and an inverse correlation with promoter methylation levels in the PE placenta. MiR-155 was found to downregulate Forkhead-box O3 (FOXO3), which in turn increased apoptosis and decreased trophoblast cell viability, migration, and invasion in in vitro experiments [110].

Researchers compiled studies on placental miRNAs and their role in the pathogenesis of PE in a rodent PE animal model [111, 112]. They found that the expression of miR-148/152 family miRNAs was upregulated in the PE placenta. This finding was verified in pregnant rats in the PE model, and Yang et al. also discovered that the downregulation of DNMT1 was accompanied by an overall increase in miR-148a/152 expression, resulting in increased expression of fatty acid binding protein 4 (FABP4) [112]. This discovery was further confirmed in HTR8 cells, where overexpression of FABP4 led to upregulation of the pro-inflammatory cytokines IL-6 and TNF-a, as well as an increase in lipid accumulation. Both in vitro and in vivo models of PE have demonstrated the involvement of miR-148a/152-FABP4 in the development of PE [112, 113].

In summary, miRNAs such as miR-34a, miR-29b, let-7a, miR-155, and miR-148a/152 have an impact on the proliferation, migration, and invasion of trophoblast cells, ultimately leading to the initiation and development of PIH (Fig. 3). Considering PIH's heterogeneity, future research efforts should focus on identifying changes in placental miRNA profiles within patient subgroups or clarifying abnormal blood and placental miRNA expression profiles among different subsets of patients with placental equivalent diseases.

#### DNA methylation and other placental genes

In the above section, we systematically summarized the latest research progress on DNA methylation involved in regulating imprinted genes, tumor suppressor genes, classical pathway-related genes, and miRNAs expression in the placenta. In addition to these genes, current research has found that DNA methylation also participates in regulating the expression of other genes in the placenta and plays an important role in the occurrence of PIH.

Activated leukocyte cell adhesion molecule (ALCAM) is an immunoglobulin cell adhesion molecule that is overexpressed in human endometrial epithelial cells and blastocysts. The stage-specific expression during embryonic development suggests that ALCAM plays a role in the initial interaction between the embryo and the maternal endometrium [114]. A decrease in ALCAM expression and an increase in DNA methylation within the ALCAM gene promoter in PE placentas, meanwhile, there was a negative correlation between ALCAM promoter methylation and its expression [91, 115]. These findings suggest that alterations in DNA methylation-mediated ALCAM expression may contribute to the development of PE by reducing trophoblast vitality and invasiveness. This is because ALCAM expression is typically observed in proliferating cells and is crucial for blastocyst implantation, thereby enhancing maternal–fetal communication [116]. During embryonic development, PAX3 (Paired Box 3) acts as a transcription factor that influences the growth of placenta. Patients with PE have a higher methylation level of the PAX3 promoter and drastically reduced levels of PAX3 in their placentas. The decreased expression of PAX3 ultimately suppresses the proliferation and invasion abilities of trophoblast cells [117].

Tyrosine 3-monooxygenase/tryptophan 5-monooxygenase activation protein, theta polypeptide (YWHAQ) is highly expressed in placental trophoblast cells and promotes trophoblast differentiation by controlling cell cycle progression [118]. Liu et al. reported that significant hypermethylation of the YWHAQ promoter in SPE placentas resulted in decreased YWHAQ and increased apoptosis of placental trophoblast cells, thereby resulting in increased levels of placental cell debris flowing into the maternal circulation [119]. This observation suggests that YWHAQ may be involved in the molecular mechanisms underlying the pathologic process of PE, but further evaluation is needed. Abnormal trophoblast cell apoptosis is implicated in the pathogenesis of PE, and endoplasmic reticulum (ER) stress is involved in the regulation of trophoblast cell apoptosis. ER oxidase 1α (ERO1α) is a glycosylated flavoenzyme that plays an important role in maintaining ER redox homeostasis. Xiong et al. reported that ERO1α expression is suppressed in trophoblast cells in the placenta of PE rats due to hypermethylation of the ERO1α promoter region [120]. These results demonstrate that promoter hypermethylation of ERO1α leads to trophoblast cell apoptosis through ER stress in the PE placentas, shedding light on the etiology of PE and potentially providing a therapeutic target for its treatment. One of the most critical steps in fertilization is the fusion of the egg plasma membrane, which is facilitated by sperm equatorial segment protein 1 (SPESP1). Yeung and Meng et al. found significant hypermethylation of the SPESP1 gene promoter as well as down-regulation of SPESP1 expression in PE placentas [91].

SERPINA3 (Serpin peptidase inhibitor clade A member 3), a serine protease inhibitor, is up-regulated in PIH placentas, which is associated with hypomethylation of the 5' region of the SERPINA3 gene [121, 122]. Overexpression of SERPINA3 in JEG-3 cells has been found to decrease cell adhesion to the extracellular matrix and neighboring cells [122]. C-Maf inducing protein (CMIP) has oncogenic properties and is expressed in a variety of malignant tumors. It was found that in PE placentas, the methylation level of the CMIP promoter was significantly reduced, and the expression level of CMIP was negatively correlated with the methylation level, which promotes trophoblast apoptosis and plays a role in the pathogenesis of PE. In addition, connective tissue growth factor (CTGF), a stroma-associated cellular protein, is a multifunctional signaling regulator involved in angiogenesis. In a study of a Chinese Han population, it was found that decreased promoter methylation resulted in increased expression of CTGF in SPE placentas, and the enhanced CTGF expression contributes to the regulation of trophoblast differentiation [123].

Aquaporin 1 (AQP1) is one of the most widely expressed water channels and plays an important role in regulating maternal–fetal fluid exchange and amniotic fluid volume [124]. It was found that AQP1 levels were elevated in PE placentas, which was associated with reduced DNA methylation in its promoter and significantly negatively correlated with the amniotic fluid index[125]. Elevated AQP1 may disrupt maternal–fetal body fluid homeostasis in PE pregnancies. In addition, guanine nucleotide binding protein (G protein) alpha 12 (GNA12) is an important regulatory molecule for signaling through transmembrane receptors. Study has shown that GNA12 expression is increased in PE placentas and hypomethylation is observed at three CpG sites of the GNA12 gene promoter [126, 127]. Death-domain associated protein (DAXX) is a histone chaperone protein that undergoes methylation changes in the DMR within the DAXX gene during CTB syncytization to STB and differentiation of in vitro cellular trophoblasts to invasive trophoblast cells. Novakovic et al. observed that loss of methylation in this DMR is associated with decreased DAXX expression [128]. However, this finding contradicts the results from the published EOPE comprehensive library of gene expression of placental villus methylation (GSE44712) [13], which states that DAXX hypomethylation leads to higher DAXX expression. Further research is needed to determine the specific role of differential DAXX expression in PE.

It is evident that trophoblast cells express a variety of proteins, and the abnormal expression of these proteins, regulated by DNA methylation, can result in abnormalities in trophoblast cell proliferation, differentiation, apoptosis, and invasion. These abnormalities may contribute to impaired placental function and the progression of PIH (Fig. 3).

### DNA methylation and placental vascular system

In the previous section, we provided a summary of the research progress on DNA methylation and its regulation of gene expression in trophoblast cells in the context of the PIH placenta. As a vascular organ, the placenta is responsible for supplying blood to both the mother and the developing baby. Therefore, it is essential for the placenta to receive an adequate blood supply. Currently, it is widely accepted that defects in trophoblast cell invasion and spiral artery remodeling during the early stages of pregnancy are the main pathological causes of placental ischemia and the initiation of PIH. However, abnormalities in the development and vascular tone of placental vessels also contribute to insufficient blood supply to the placenta, ultimately leading to placental ischemia and dysfunction. In this section, we aim to summarize the current knowledge and provide a comprehensive evaluation of aberrant DNA methylation of genes associated with vascular development and function in PIH placentas (Table 1).

#### DNA methylation and placental angiogenesis, vascular remodeling

PIH is a placental vascular pathology, and hypoxia is known to influence placental angiogenesis. The oxygen-sensitive transcriptional activator HIF1 is a key mediator of the transcriptional response to hypoxic conditions. The HIF1 pathway has been identified as a master regulator of angiogenesis and is involved in vasculature formation through synergistic interactions with other proangiogenic factors such as vascular endothelial growth factor (VEGF), placental growth factor (PlGF), and angiopoietins [129]. Recently, Kaur et al. found that placental DNA methylation and expression of HIF1α and 3α genes are altered and associated with PIH, and birth outcomes [130]. Furthermore, DNA methylation in the promoter region of HIF1α was found to be negatively correlated with its expression levels in PIH placentas. Considering the significant role of HIF1 in angiogenesis and vasculogenesis, it should be considered a promising target for the treatment of PIH. VEGF is a crucial mediator of angiogenesis in both physiological and pathological processes. Sundrani et al. discovered that the methylation of the VEGF gene promoter was significantly reduced in early preterm PE placentas, while its expression was significantly increased. Disruptions in the levels of VEGF and its receptor can impair angiogenesis, resulting in placental insufficiency and endothelial dysfunction [131]. This finding highlights the significance of altered DNA methylation in placental angiogenesis and adverse pregnancy outcomes. Factors related to angiogenesis, such as soluble FMS-like tyrosine kinase-1 (sFlt-1) and PlGF, play significant roles in placental angiogenesis and vasculogenesis. Dave et al. observed hypomethylation at several CpG sites in the promoters of PlGF and Flt-1 genes in PE placentas. DNA methylation at different CpG sites was correlated with the two genes expression and was associated with maternal blood pressure [132].

Extracellular matrix (ECM) plays a crucial role in vascular remodeling during pregnancy. This process of trophoblasts invading the endometrial stroma, forming the myometrium and the deep layer of the placenta, is essential for fetal development [133]. Matrix metalloproteinases (MMPs), a disintegrin and metalloproteinases (ADAMs), and a disintegrin and metalloproteinase with thrombospondin motifs (ADAMTs) are all examples of MMPs that are secreted from placental cells during this process [134, 135]. MMPs and their tissue inhibitors (TIMPs) play crucial roles in the trophoblast's proper invasion into maternal tissue. An imbalanced expression of MMPs and TIMPs impairs proper placentation, and DNA methylation may affect their expressions [136, 137]. The aberrant expressions of these proteins are tightly regulated by DNA methylation, which can impair the ECM remodeling process during pregnancy. In PE placentas, several genes, including MMP9, TIMP3, and ADAMTS7, were significantly less methylated within the gene promoter. Abnormal expression of MMP9 can increase the risk of developing PE by preventing adhesion molecule-mediated invasion of the EVTs [138]. Wang et al. found significantly higher expression levels of MMP9 in PE placentas, and this increased expression was well correlated with promoter demethylation [136].

TIMPs are present in a 1:1 stoichiometric ratio with MMPs in tissues, where they locally and temporarily suppress MMP activity [139]. TIMP3 is abundantly expressed in the placenta and plays a critical role in controlling placental vascular remodeling during placentation [139]. Yuen et al. confirmed significant hypomethylation of the TIMP3 promoter in EOPE placentas [140]. Xiang et al. discovered an inverse correlation between hypomethylation of the TIMP3 promoter and TIMP3 expression in PE placentas [141]. Similarly, Cruz et al. found hypomethylation of the TIMP3 gene promoter, as well as increased TIMP3 expression in corresponding placental samples from PE, EOPE, and LOPE compared to controls [142]. These findings demonstrate the significant role of DNA methylation in controlling TIMP3 expression. The hypomethylation of the TIMP3 promoter increase TIMP3 expression, leading to decreased trophoblast invasiveness during placental development and contributing to low placental perfusion in PE. Therefore, TIMP3 may serve as a prenatal marker for the development of PE. Additionally, abnormal expressions of some members of the ADAMTS family have been associated with PE progression [143]. Zhang et al. discovered that hypomethylation of the ADAMTS7 gene promoter resulted in higher expression of ADAMTS7 in PE placentas [144]. These findings suggest a role for ADAMTS7 in the pathophysiology of PE, as it can inhibit the migration, invasion, and survival of trophoblast cells in vitro, leading to inadequate repair and blood flow in the placental vascular spiral artery.

#### DNA methylation and placental vascular function

After placentation in early pregnancy, adequate blood flow in the placenta is vital for maintaining placental function. The blood arteries in the placenta lack their own neural innervation. Therefore, local synthesis of vasoactive chemicals and vascular regulators are essential for regulating vascular activity, function, and blood flow within the placenta [145, 146].

Arginine vasopressin (AVP), a traditional vasoconstrictor, controls vascular tone by binding to its receptors (primarily V1a (AVPR1A), V1b (AVPR1B), and V2 (AVPR2) subtypes) in vascular smooth muscle cells (SMCs). Gao et al. were the first to study the role of AVP in the placental vascular system and discovered that placental vessels had a diminished sensitivity to AVP-induced constriction in PE placentas. They also observed downregulation of AVPR1A and protein kinase C isoform β (PKCβ), as well as a dramatic increase in promoter DNA methylation within the two genes in the PE placental vessels [147]. This study revealed that aberrant DNA methylation-mediated gene expressions are correlated with vascular dysfunction in the PE placental circulation. Oxytocin (OXT), another traditional vasoconstrictor, is predominantly known as a potent stimulator of uterine smooth muscle and plays an important role in maintaining placental circulation [148]. The actions of OXT are mediated through its receptor (OXTR). In the context of pregnancy, OXT and OXTR are abundant in the human placenta. Oxytocin is increased in the placental system during late pregnancy and the onset of labor, and it exhibits concentration-dependent contractions in the placental vascular system [149]. DNA hypermethylation of CpG islands in the OXTR gene promoter leads to the transcriptional inactivation of the OXTR gene. This is why Fan et al. found that OXT-induced vasoconstriction was significantly reduced in the PE placental vessels [150].

Additionally, ROS can regulate vascular constriction and relaxation. NADPH oxidases (NOX) catalyze the production of ROS and regulate redox-dependent processes in various biological contexts. Recently, Yeung et al. reported that DNA hypermethylation within the promoter of the NOX5 gene were observed in the PE placentas [91]. Given the importance of the NOX/ROS pathway in regulating vascular tone, this finding suggests that DNA methylation of the NOX5 gene promoter may play a role in placental vascular dysfunction in PE placentas. As an important vascular mediator, the large-conductance Ca^2+^-activated K^+^ channel (BK) is a tetramer composed of a pore-forming alpha subunit (BKα) and up to four accessory beta subunits (BKβ). BK plays a crucial role in determining vascular tone. Chen et al. reported that placental hypoxia/ischemia during pregnancy inhibits pregnancy-induced demethylation of the BKβ1 gene and upregulation of BKβ1 expression in uterine arteries. They presented new insights into the role of DNA methylation in the association of gestational hypoxia/ischemia with aberrant uteroplacental circulation and an increased risk of PE [151].

In summary, whether it is angiogenesis and vascular remodeling during placentation or abnormal regulation of placental vascular function during mid-to-late pregnancy, it will affect placental function and lead to placental-related diseases such as PIH. Despite the aforementioned research (Table 1), there is currently a lack of data on DNA methylation of genes related to the placental vascular system in PIH placentas. Further in-depth research is urgently needed on the roles of DNA methylation in placental vascular angiogenesis, remodeling, and function regulation, as well as its role in the occurrence of PIH.

## DNA methylation changes in PIH maternal blood

Researchers were able to identify a high number of DMRs that could be used as early diagnostic markers for PIH using maternal peripheral blood DNA [152, 153]. For example, three neuronal genes—GABRA1, BEX1, and GRIN2B—were significantly differentially methylated and expressed at low levels in the maternal blood, suggesting a correlation with seizure episodes of PIH patients, as determined by whole-genome methylation analysis [153]. Currently, whole-genome bisulfite sequencing was used on maternal blood to elucidate the methylation pattern associated with PIH, leading to the identification of new candidate risk genes. Additionally, these potential markers may have application in the early diagnosis of PE (Table 2).

**Table 2:** DNA Methylation Changes in PIH Maternal Blood

Sample size	Gestational age (weeks)	Sample type	DNA methylation	Target gene	Gene expression	Ref
22	7–41	Plasma	Hypermethylation	RASSF1A	Decreased	[160]
25	35.88 ± 2.65	Plasma	Hypomethylation	Maspin	Increased	[165]
48	35.39 ± 3.33	Plasma	Hypomethylation	TIMP3	Increased	[141]
-	35.3 ± 2.5	Plasma	Hypermethylation	STAT5A	Decreased	[166]
50	-	Plasma	Hypomethylation	GNA12	Increased	[127]
90	34.11 ± 4.83	Plasma	Hypomethylation	CTGF	Increased	[123]
21	35.4 ± 0.9	Plasma	Hypomethylation	MMP1	Increased	[170]
22	28–38	Plasma	Hypomethylation	TBXAS1	Increased	[174]
45	32.0 ± 6.5	Plasma	Hypomethylation	IL17	Increased	[176]

*RASSF1A* RAS association domain family 1 A, *TIMP3* tissue inhibitors of metalloproteinase-3, *STAT5A* signal transducer and activator of transcription 5A, *GNA12* guanine nucleotide binding protein (G protein) alpha 12, *CTGF* connective tissue growth factor, *MMP1* Matrix metalloproteinase 1, *TBXAS1* thromboxane A synthase 1, *IL17* interleukin-17

### DNA methylation and maternal plasma

Pregnancy-related problems are associated with cell-free DNA (cfDNA) and cell-free fetal DNA (cffDNA) in maternal plasma [154, 155]. Total cfDNA levels are significantly higher in late pregnancy for pregnant women with PE [156]. This discovery opens the door for the analysis of cfDNA in maternal plasma to be used in clinical settings for the detection and monitoring of PE.

Recent research has proposed the use of cfDNA methylation and transcriptome indicators in early pregnancy to predict the onset of PE before the manifestation of clinical symptoms [157]. For example, RAS association domain family 1A (RASSF1A) DNA sequences were detected in the mother's blood; the gene was silenced and frequently inactivated by hypermethylation of the promoter region; and reduced RASSF1A expression was positively correlated with the severity of PE [158–160]. Rahat et al. concluded that there is an association of DNA methylation of the RASSF1A promoter and RASSF1A expression in PE maternal plasma, and the increased RASSF1A expression contributes to the development of PE [161]. This finding suggests that RASSF1A could be utilized as a non-invasive diagnostic tool for PE. Building upon these findings, Salvianti et al. prospectively assessed RASSF1A cfDNA in maternal plasma during pregnancy, specifically examining the methylation status of the promoter gene sequence. They found that total cfDNA and cffDNA were sufficiently predictive of PE in high-risk women compared with non-PE high-risk women between 17 and 30 weeks of gestation, and that cfDNA and cffDNA could be quantified with hypermethylated RASSF1A, suggesting that hypermethylated RASSF1A could provide evidence as a reliable marker for PE [162]. Apart from RASSF1A, DSCR3 (Down syndrome critical region gene 3) may also serve as an epigenetic marker for hypermethylated cffDNA. After 24–32 weeks, the concentration of cffDNA (DSCR3 and RASSF1A) significantly increased in patients with EOPE [163]. However, there are few current studies on DSCR3, and it is hoped that more subsequent studies will delve deeper into DSCR3 to determine whether it can be a biomarker.

Maspin is a well-known tumor suppressor gene. In PE plasma samples, the level of its promoter methylation exhibits further downregulation [164]. Unmethylated maspin DNA levels in the blood are 5.5 times higher in pregnant women with SPE compared to normal pregnant women in the third trimester. However, there is no statistically significant difference between moderate PE patients and normal pregnant women [165]. The methylation pattern of the TIMP3 promoter is similar to that of maspin. It is moderately methylated in normal placental tissue and almost completely methylated in maternal blood DNA. The level of unmethylated TIMP3 sequence is significantly higher in PE maternal plasma. This is consistent with the lower methylation level of the TIMP3 promoter in PE placentas [141]. Yuen et al. reported that gene-specific hypomethylation may be a common phenomenon in EOPE plasma [140]. Therefore, the alteration of unmethylated TIMP3 sequences in maternal plasma can be utilized to predict PE. An increase in the amount of unmethylated TIMP3 cfDNA was taken into account, which could improve the sensitivity of non-invasive screening for PE pregnancies.

STAT5A (signal transducer and activator of transcription 5A) is a transcription factor with well-known anti-apoptotic role. Its expression level in placental villi is highest in the first trimester and decreases over the remainder of pregnancy [166]. The pattern of promoter methylation of STAT5A in maternal plasma cfDNA in pregnant women with PE is consistent with that in placental villi, which are elevated. This suggests that it is a useful epigenetic marker in women with PE [166].Ye et al. investigated the potential association between the methylation of the GNA12 promoter and PE [127]. The methylation level at three CpG sites of the GNA12 promoter was significantly lower in both the PE placental and peripheral blood DNA samples. Consistent with the decreased methylation level, the expression level of GNA12 was higher in PE patients. These results showed that PE is associated with decreased methylation of the GNA12 promoter, which can be used as an early diagnostic biomarker of PE [127]. In addition, higher CTGF expression in trophoblast cells is associated with a reduced frequency of CTGF promoter methylation, which is observed in the peripheral blood of PE pregnant women compared to controls [123]. Evidence from the Han Chinese population indicates a link between PE and poor methylation of the CTGF promoter in the peripheral blood [123]. Nonetheless, more evidence is needed to establish CTGF as a gene factor appropriate for wide-scale application in early PE detection, as the sample size was very small.

Although it has been found that abnormal DNA methylation of genes in cfDNA and cffDNA can be used as markers for the prenatal diagnosis of PIH, more rigorous research is required to confirm the presence of cfDNA and cffDNA changes in the plasma of PE patients and establish a correlation between these changes and the placenta and maternal circulation. Furthermore, further investigation into DNA methylation is necessary to identify molecules that are abnormally produced in maternal plasma and determine their role in the etiology of PE.

### DNA methylation and maternal vascular function

Among the hallmarks of PE is vascular dysfunction in the mother. In PE maternal vessels, 65 genes are found to be hypomethylated, and there is evidence of overexpression of 75 molecular functions and 149 biological processes. These processes and functions include those involved in smooth muscle contraction, thrombosis, inflammation, redox homeostasis, and amino acid metabolism [167]. The most recently documented maternal vascular phenotypes in PE are the overexpression of MMPs in endothelial cells, vascular SMCs, and invading neutrophils [168, 169]. In PE patients, the great omental artery has been found to have significantly lower levels of methylation in the promoter regions of the MMP1 and MMP8 genes in neutrophils, which is strongly correlated with elevated expression [170]. As mentioned earlier, overexpressed MMP1 exerts its many biological functions through protease-activated receptor-1 (PAR1). Since PAR1 is only expressed during pregnancy, its activation leads to the translocation of TET2 and nuclear factor-kappa B (NF-κB) from the cytoplasm to the nucleus in neutrophils, resulting in increased expression of inflammation-related genes and a more rapid progression of PE [171].

A decrease in prostacyclin, a vasodilator and platelet inhibitor, and an increase in thromboxane, an effective vasoconstrictor and platelet activator, have been reported in the PE placentas. These changes have subsequently been confirmed in maternal blood and urine [172, 173]. Thromboxane A2 is produced by thromboxane A synthase 1 (TBXAS1), which is responsible for the isomerization of prostaglandin H2 (a precursor of both thromboxane and prostacyclin). Mousa et al. confirmed a strong correlation between decreased DNA methylation in the TBXAS1 gene promoter region and increased TBXAS1 expression in the great omental arteries of women with PE [174]. Increased TBXAS1 expression was also observed in maternal endothelial cells, vascular SMCs, invading neutrophils, and white blood cells of women with PE [174]. Furthermore, hypertension and chronic inflammatory illnesses are also linked to the cytokine family, which is a potent inflammatory agent [175]. Walsh et al. found significantly reduced DNA methylation of the interleukin-17 (IL17) gene promoter in omental arteries of PE women and demonstrated that IL17 levels are regulated by DNA methylation, and increases in the PE circulation [176].

In summary, these investigations demonstrate that DNA methylation is important in regulating maternal vascular function and contributes to the development of PIH. Clinical diagnosis and therapy of PIH are aided by the information provided by these studies, and the pathophysiology of this condition is better understood.

## DNA methylation changes in PIH umbilical cord

### DNA methylation in umbilical cord blood

Currently, only a small number of studies have investigated the variations in local or global DNA methylation in umbilical cord blood cells of newborns with PIH [177, 178]. Kazmi et al. detected the levels of global DNA methylation in cord blood among neonates with PIH and PE and found that the epigenome-wide correlation of PIH with offspring DNA methylation was moderately consistent with the equivalent epigenome-wide correlation of PE with offspring DNA methylation [178]. This demonstrates the presence of numerous DNA methylation markers in cord blood in cases of PIH and PE. Ching et al. initially examined DNA methylation throughout the entire genome in cord blood from neonates with EOPE and indicated that the DNA methylation pattern of a considerable number of genes changed in EOPE cord blood [179]. They found that promoters of 643 and 389 genes were hypomethylated and hypermethylated, respectively. Genes in the farnesoid X receptor and liver X receptor (FXR/LXR) pathways were enriched, indicating dysfunction of lipid metabolism in EOPE cord blood cells. Additionally, a group of genes involved in inflammation, lipid metabolism, and proliferation were persistently differentially methylated in EOPE cord blood cells [179]. These findings provide evidence of prominent DNA methylation modifications in cord blood DNAs associated with EOPE and suggest a connection between inflammation and lipid dysregulation in EOPE-associated newborns and a higher risk of cardiovascular diseases later in adulthood.

Leptin is a hormone that plays an important role in maintaining healthy cardiac metabolism and stable energy levels. Study has shown that in the cord blood of pregnant women with PE, there is reduced methylation of the leptin gene at the CpG14 locus and increased leptin level, which negatively affected maternal–fetal nutrient exchange and placental function [180]. HIF3A is a member of the HIF gene family and is thought to play an important role in vascular development, and metabolism. Mansell et al. first found that PE was associated with increased HIF3A expression due to hypomethylation of the HIF3A gene promoter in umbilical cord blood, which was consistent with hypoxia-induced up-regulation of HIF3A transcription [181]. However, further studies are needed to determine the underlying mechanisms.

Mitochondria are the energy factories of the cell; therefore, it is necessary to study the expression and activity of mitochondrial genes in cord blood cells. Novielli et al. first characterized mitochondrial DNA in cord blood from PE and found that DNA methylation levels of the D-loop (mitochondrial loci relevant to replication) gene were decreased and its expression increased in PE cases [182]. Low D-loop methylation levels are associated with poorer fetal prognosis, and this association is particularly evident in PE cases, where D-loop methylation levels are further reduced and strongly positively correlated with fetal weight and umbilical vein pO2 [182]. In the aforementioned placenta chapter, it is mentioned that the expression and activity of 11βHSD2 are reduced in the PE placenta, leading to increased exposure of the fetus to glucocorticoids. Infants born to mothers with PE have been found to have decreased levels of 11βHSD2 promoter methylation in cord blood, indicating a favorable association between 11βHSD2 promoter methylation and PE, as well as a possible increase in 11βHSD2 expression in PE offspring [183]. More research is required to understand the role that hypomethylation of the 11βHSD2 promoter plays in the progression of diseases in the PE offspring.

In conclusion, these investigations on cord blood have demonstrated the importance of DNA methylation in cord blood and its contribution to the development of PIH (Table 3), which has negative consequences for both the mother and the fetus. The information provided by these studies aids in the clinical diagnosis and therapy of PIH, and enhances our understanding of the pathophysiology of this condition.

**Table 3:** Changes in DNA methylation in umbilical cord and in offspring of PIH cases

Sample size	Sample type	DNA methylation	Target gene	Gene expression	Functions	Ref
877	umbilical cord blood	Hypomethylation	Leptin	Increased	Reduce maternal–fetal nutrient exchange	[180]
938	umbilical cord blood	Hypomethylation	HIF3A	Increased	Inhibition of vascular development	[181]
17	umbilical cord blood	Hypomethylation	D-loop	Increased	Lead to poorer fetal prognosis	[182]
40	umbilical vein	Hypermethylation	AVPR1a、OXTR	Decreased	Decrease contractility of the umbilical vein	[185]
5	offspring	Hypermethylation	ARID1B	Decreased	Increase risk of cardiovascular disease in offspring	[196]
5	offspring	Hypomethylation	CTHRC1	Increased	Increase risk of cardiovascular disease in offspring	[196]

*HIF* hypoxia inducible factor, *D-loop* mitochondrial loci relevant to replication, *ARID1B* AT-rich interacting structural domain 1B, *CTHRC1* collagen-containing triple helical repeat 1

### DNA methylation in umbilical cord vessels

Umbilical vein plays a crucial role in the circulation between the placenta and the fetus, as it carries oxygenated and nutrient-rich blood from the placenta to the fetus. 5hmC is produced from 5mC and promotes DNA methylation plasticity through oxidation by the TET enzyme. 5hmC is not only an intermediate modification in the active demethylation process of DNA, but also an important epigenetic marker for neurodevelopment, and embryonic stem cell differentiation [184]. Sun et al. discovered that in patients with PE, both TET2 expression and 5hmC levels were significantly reduced in the umbilical veins [11]. Additionally, a decreased sensitivity to vasoconstriction of AVP and OXT was observed in the umbilical vein of PE patients, which has been linked to hypermethylation of AVPR1a, OXTR, and PKCβ. This is because hypermethylation of these genes leads to a relatively low gene expression pattern in the umbilical vein of PE patients [185].

Endothelial colony-forming cells (ECFCs) have been found to have a decreased number and compromised activities in PE umbilical cord [186]. ECFCs are a relatively homogeneous subset of late-growth endothelial progenitor cells that possess the ability to multiply and migrate to the site of vascular formation, directly contributing to endothelial development [187]. Brodowski et al. conducted a study on the methylation pattern of ECFCs in the umbilical cord of PE cases. They observed a differential methylation pattern of umbilical cord ECFCs in PE compared to normal pregnancies, with 1266 CpG sites showing differences in passage 3 and 2362 sites in passage 5. Moreover, 1260 of the PE-related methylation changes detected in passage 3 were confirmed in passage 5, suggesting that the methylation modifications induced by PE are stable [188]. Further research is necessary to comprehend the causes of DNA methylation alterations in PE umbilical cord blood and vessels, as well as the potential effects of these changes on the health of future generations.

The placenta plays a significant role in the deterioration of the mother's cardiovascular system during pregnancy. Throughout pregnancy, DNA methylation patterns can shift unexpectedly, occurring in the placental, maternal, and fetal sides. Therefore, it is crucial to detect maternal and fetal blood to investigate placental diseases. The availability and utilization of cfDNA and cffDNA in obstetrics have provided opportunities for non-invasive prenatal diagnosis of PIH.

## DNA methylation changes in PIH neonates and children

Strong evidence suggests that the timing of development from conception to infancy is influenced by interactions between the genome and the environment, which can have long-lasting effects on offspring [189–191]. Conditions such as obesity, hypertension, and poor cardiometabolic markers in children have been associated with these conditions in the mother [191, 192]. Epigenetic imprinting, particularly DNA methylation, occurs during development, allowing the environment to not only influence gene expression but also impact future generations [191, 193]. This implies that DNA methylation play a crucial role in regulating the physiopathology of PIH in both the fetus and postnatal stages (Table 3).

The variations in DNA methylation in the blood, placenta, and umbilical cord of PIH mothers, as discussed earlier, may have effects on the developing fetus. Recent clinical studies in neonates and children have confirmed this. A few years ago, Suzuki et al. conducted DNA methylation analysis of fetal exposure to PE using amniotic membrane samples from delivered placentas [194]. They identified different DMRs between amniotic epithelial cells and mesenchymal stromal cells, confirming that exposure to PE alters fetal DNA methylation. This study also supported the use of amniotic membranes as a substitute for the fetus to address genome-wide DNA methylation. Several cohort studies have reported an increased risk of cardiovascular disease and pulmonary vascular malfunction in adults who were born to mothers with PIH [195, 196]. For example, Julian et al. found that offspring of women with PIH had higher pulmonary artery systolic pressure compared to controls [196]. To determine whether DNA methylation of genes involved in the regulation of vascular function is present in offspring of women with PIH, they compared DNA methylation and gene expression patterns in peripheral blood mononuclear cells from young male offspring of PIH women with normotensive controls [196]. They found that three genes, namely collagen triple helix repeat containing 1 (CTHRC1), AT rich interactive domain 1B (ARID1B), and SPARC related modular calcium binding 2 (SMOC2), had six different DMRs in the offspring of patients with PIH. Among these genes, ARID1B had impaired gene expression, and CTHRC1 tended to be higher. The transcriptional activity of the two genes was inversely proportional to their DNA methylation status [196].

Based on these findings, it is speculated that DNA methylation may play a role in mediating the increased risk of vascular disease later in life associated with PIH. Due to ethical constraints on human research, experimental animals were used to investigate the effects of maternal PIH on gene DNA methylation in multiple generations of offspring and its role in the occurrence of offspring diseases [197, 198]. For instance, Guan et al. [197] found that DNA hypomethylation of the G signaling gene in F1 sperm and F2 thoracic aorta, along with hyperactivation of downstream PI3K-Akt signaling, suggests that DNA methylation plays a crucial role in the development of hypertension in PIH offspring. Since PIH is a "major obstetric syndrome" involving multiple pathological processes that can overlap and ultimately stimulate the same pathway, such as endothelial activation, intravascular inflammation, and syncytial trophoblastic stress, it is important to investigate the link between intergenerational transmission and DNA methylation in a precise PIH animal model.

In conclusion, existing clinical and animal experiments have shown that alterations in DNA methylation, induced by PIH, are linked to unfavorable outcomes in children and offspring following birth. These outcomes include an elevated susceptibility to cardiovascular and metabolic diseases in PIH offspring. However, due to the complex nature of PIH pathogenesis, there is still a significant knowledge gap in this field. Therefore, it is imperative and essential to conduct further research in this area.

## Conclusion and outlook

Pregnancy offers a unique opportunity to investigate stress and stress responses. In the case of pathological pregnancies, understanding epigenetic mechanisms, particularly DNA methylation, may assist in identifying new biomarkers associated with disease risk related to pregnancy. These biomarkers could be crucial for developing tools to detect risk factors and exposure levels at an early stage. In this review, we summarize the latest research progress on the role of DNA methylation in the pathophysiology of PIH. This review supports the notion that many genes involved in the initiation and development of PIH, mediated by DNA methylation, could serve as diagnostic markers for the disease. Moreover, addressing DNA methylation may prove to be a valuable therapeutic strategy for PIH.

This review focuses on three major aspects: maternal, fetal, and offspring, and provides a comprehensive account of the relationship between DNA methylation and the trophoblastic function of the placenta, genes related to the placental microenvironment, the placental vascular system, maternal blood and vascular function, abnormalities of umbilical cord blood and vascular function in the genesis and development of PIH, as well as the alteration of DNA methylation in the offspring of PIH. By doing so, our aim is to enhance understanding of the dynamic biological processes underlying the development of PIH. These insights will assist clinicians in recognizing adverse pregnancy outcomes caused by PIH and tailoring interventions to reduce risks, thereby promoting healthy pregnancies for all mothers. However, for most of the current studies, DNA methylation is usually identified in whole tissue/blood and there is some heterogeneity in these samples in terms of cell type, which can be a strong limitation.

The present study provides important information in understanding and exploring the epigenetic mechanisms of PIH pathogenesis. It is clear that changes in DNA methylation may play a crucial role in the development of PIH and that changes in DNA methylation play a key role in the screening, diagnosis and treatment of PIH. However, larger and more robust studies are needed to strengthen the existing understanding, and further research on DNA methylation must continue to provide a comprehensive picture of the epigenetic landscape. This review aims to serve as a catalyst for future investigations into the role of DNA methylation in the development of PIH, and hopes to inspire academics to delve deeper into this topic.

## Data Availability

No datasets were generated or analysed during the current study.

## Abbreviations

PIH: Pregnancy-induced hypertension
PE: Preeclampsia
SPE: Severe preeclampsia
EOPE: Early-onset preeclampsia
LOPE: Late-onset preeclampsia
EOSPE: Early-onset severe preeclampsia
DM: Differential methylation
DMRs: Differential methylation regions
DNMT: DNA methyltransferase
CTB: Cytotrophoblast
STB: Syncytiotrophoblast
EVT: Extravilloustrophoblast
TET: Ten–eleven translocation
MMP: Matrix metalloproteinase
TIMP: Tissue inhibitors of metalloproteinase
IGF: Insulin-like growth factor
HIF: Hypoxia inducible factor
