# Correction: DNA methylation landscape in pregnancyinduced hypertension: progress and challenges

**DOI:** 10.1186/s12958-024-01270-2

**Published:** 2024-08-07

**Authors:** Fengying Deng, Jiahui Lei, Junlan Qiu, Chenxuan Zhao, Xietong Wang, Min Li, Miao Sun, Meihua Zhang, Qinqin Gao

**Affiliations:** 1 Key Laboratory of Maternal & Fetal Medicine of National Health Commission of China, Shandong Provincial Maternal and Child Health Care Hospital Affiliated to Qingdao University, Jinan, 250014 China; 2 Institute for Fetology, The First Affiliated Hospital of Soochow University, Suzhou, 215006 P. R. China; 3 Department of Oncology and Hematology, Suzhou Hospital, Affiliated Hospital of Medical School, Nanjing University, Suzhou, Jiangsu 215153 P.R. China; 4 Department of Obstetrics and Gynecology, The First Affiliated Hospital of Soochow University, Suzhou, 215006 P. R. China

**Correction: Reprod Biol Endocrinol 22**,** 77 (2024)**

10.1186/s12958-024-01248-0

Following publication of the original article [1], the authors reported an error to the authors’ affiliations. Affiliation 1 was accidentally removed from authors Fengying Deng, Jiahui Lei and Chenxuan Zhao during the proofing stage. Affiliation 1 “Key Laboratory of Maternal & Fetal Medicine of National Health Commission of China, Shandong Provincial Maternal and Child Health Care Hospital Affiliated to Qingdao University, Jinan 250014, China” has been added back to authors Fengying Deng, Jiahui Lei and Chenxuan Zhao.

The original article [1] has been updated.
